# Using data linkage to electronic patient records to assess the validity of selected mental health diagnoses in English Hospital Episode Statistics (HES)

**DOI:** 10.1371/journal.pone.0195002

**Published:** 2018-03-26

**Authors:** Katrina Alice Southworth Davis, Oliver Bashford, Amelia Jewell, Hitesh Shetty, Robert J. Stewart, Cathie L. M. Sudlow, Matthew Hugo Hotopf

**Affiliations:** 1 King's College London, Department of Psychological Medicine, Institute of Psychiatry Psychology and Neuroscience, London, United Kingdom; 2 South London and Maudsley NHS Foundation Trust, National Institute for Health Research Biomedical Research Centre, De Crespigny Park, Denmark Hill, London, United Kingdom; 3 Surrey and Borders Partnership NHS Foundation Trust, Surrey, United Kingdom; 4 UK Biobank and Centre for Clinical Brain Sciences, University of Edinburgh, Edinburgh, United Kingdom; Karolinska Institutet, SWEDEN

## Abstract

**Background:**

Administrative data can be used to support research, such as in the UK Biobank. Hospital Episode Statistics (HES) are national data for England that include contain ICD-10 diagnoses for inpatient mental healthcare episodes, but the validity of these diagnoses for research purposes has not been assessed.

**Methods:**

250 peoples' HES records were selected based on a HES recorded inpatient stay at the South London and Maudsley NHS Foundation Trust with a diagnosis of schizophrenia, a wider schizophrenia spectrum disorder, bipolar affective disorder or unipolar depression. A gold-standard research diagnosis was made using Clinical Records Interactive Search pseudonymised electronic patient records using, and the OPCRIT+ algorithm.

**Results:**

Positive predictive value at the level of lifetime psychiatric disorder was 100%, and at the level of lifetime diagnosis in the four categories of schizophrenia, wider schizophrenia spectrum, bipolar or unipolar depression was 73% (68–79). Agreement varied by diagnosis, with schizophrenia having the highest PPV at 90% (80–96). Each person had an average of five psychiatric HES records. An algorithm that looked at the last recorded psychiatric diagnosis led to greatest overall agreement with the research diagnosis.

**Discussion:**

For people who have a HES record from a psychiatric admission with a diagnosis of schizophrenia spectrum disorder, bipolar affective disorder or unipolar depression, HES records appear to be a good indicator of a mental disorder, and can provide a diagnostic category with reasonable certainty. For these diagnoses, HES records can be an effective way of ascertaining psychiatric diagnosis.

## Introduction

Mental health research using data derived from clinical records and administrative data can be highly informative.[1–4] There are benefits of using records to enrich research cohorts with variables such as hospital admissions, diagnoses and medication use.[5] The use of administrative data to ascertain diagnoses is an efficient means to follow participants in cohort studies. However, in a recent systematic review on the validity of psychiatric diagnoses in administrative data we showed that there were large differences in the validity of diagnoses between data sources.[6] We concluded that researchers should conduct validation studies on the datasets they proposed using to guide interpretation.

**Fig 1:**
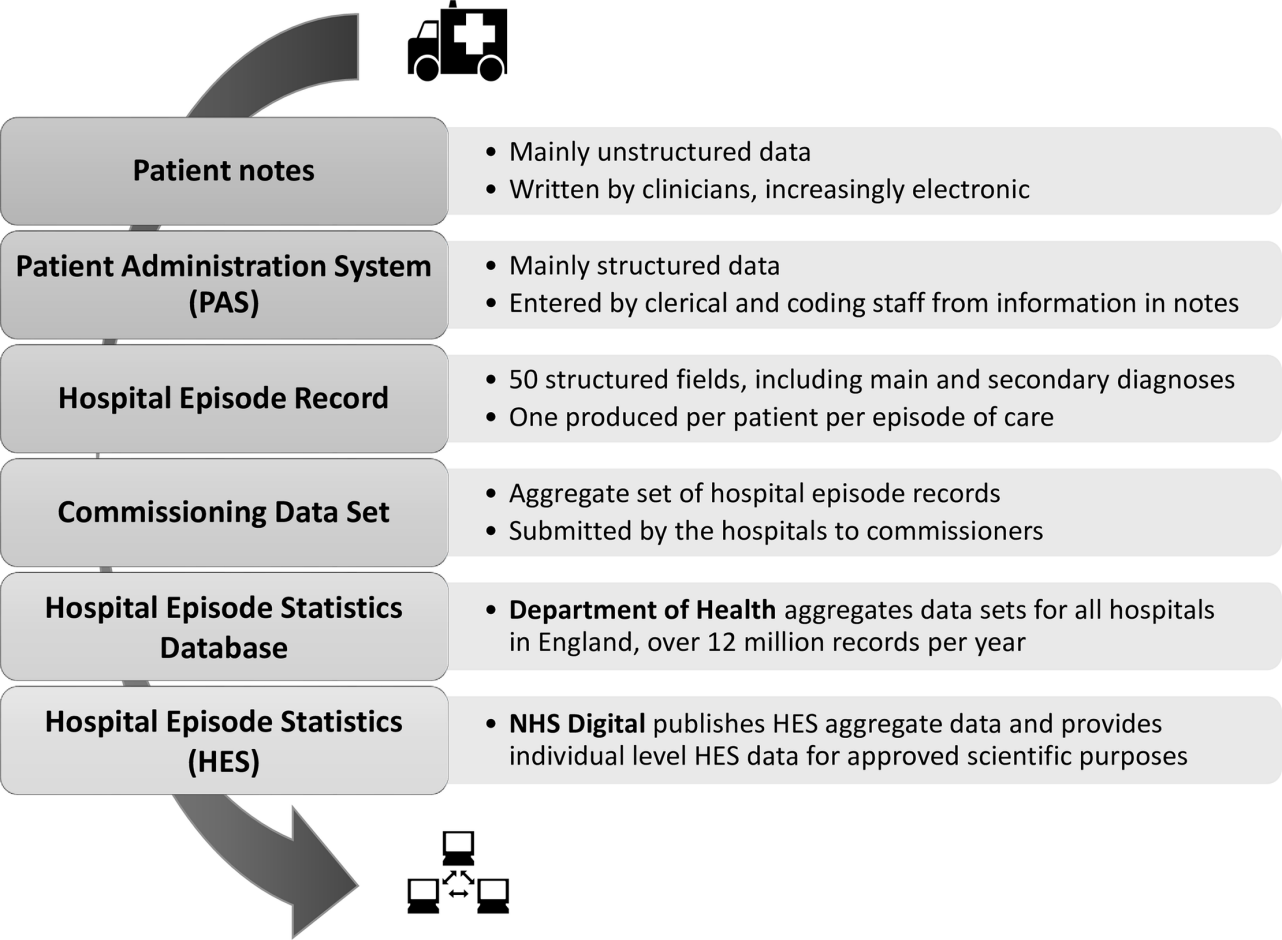
Inpatient Hospital Episode Statistics pathway from patient to researcher.

We present a validation of English National Health Service (NHS) Hospital Episode Statistics' (HES) diagnostic codes for psychiatric admissions. HES is an administrative data resource which provides records of hospital admissions, outpatient and accident and emergency department visits for individuals receiving NHS hospital treatment in England. Similar systems are available in Scotland and Wales. HES data are widely used in research, including mental health research.[7–9] Mental health providers have contributed HES inpatient data since 1996. Fig 1 shows how HES inpatient data are assembled from full text patient records via a coded aggregate dataset that represents the activity per hospital, and then combined to capture all hospital activity in England.[10] NHS Digital manage access to HES, which includes publishing regular aggregate data and managing access to individual patient-level data, including linkages to research datasets.[11]

Audits of inpatient HES from general hospitals have demonstrated that diagnosis can be unreliable [12] but no audit of HES diagnosis has been conducted for mental health providers. The motivation for this validation study was primarily to inform research on mental health disorders conducted using data from UK Biobank. This research resource recruited 500,000 people aged between 40–69 years in 2006–2010 from across the UK [13] who agreed to have their health followed through linkages to health-related records, which include HES. We aimed to investigate the accuracy and reliability of mental health diagnoses in HES inpatient records from a mental health provider, to produce an external validation for schizophrenia spectrum and affective disorders diagnoses. Since it has been shown that the choice of algorithm for extracting psychiatric diagnosis from administrative datasets can have substantial impact on the accuracy of the diagnoses derived [14–16] we aimed to provide a range of accuracy statistics for possible algorithms, to enable future researchers to evaluate and choose the algorithms most suited to their purposes.

## Materials and methods

### Data source

The South London and Maudsley (SLaM) NHS Foundation Trust provides comprehensive NHS mental health services for a defined geographic catchment of around 1.2 million residents in South London, along with a number of smaller specialist tertiary referral centres. SLaM introduced an electronic records system across all its services from 2006. The Clinical Record Interactive Search (CRIS) system and its associated governance structures were developed to allow approved researchers to interrogate fully de-identified electronic health records, and has been used to provide a SLaM case register.[17, 18] A CRIS records includes the entirety of the electronic patient record from the NHS Trust. This includes structured fields such as age and ethnicity, forms such as for care planning and any detentions under mental health law, free text such as clinical notes and clerkings, and attachments of correspondence such as letters to primary care physicians and onward referrals. Changes and additions in the patient record are updated to CRIS on a daily basis to maintain it as a contemporary source.

Linkage of the CRIS/SLaM register with other databases is managed through the SLaM Clinical Data Linkage Service (CDLS). Linkage to HES is carried out by NHS Digital using NHS numbers, which are unique patient identifiers. A record in the CRIS/SLaM register will have linked HES records that include admissions to SlaM, to other mental health providers in England, and to general hospitals.

Research using the CRIS system, based on SLaM electronic records and the associated linkages has the endorsement of a patient-led oversight group. CRIS/SLaM has approval for analysis as a source of secondary data from the Oxford Research Ethics Committee C (reference 06/H0606/71+5) with access to restricted to researchers holding an honorary or substantive contract with SLaM.[17]

### Data extract for this study

We studied HES records generated by SLaM in 2008–2013 where the primary diagnosis was schizophrenia (F20), wider schizophrenia spectrum disorder (F21-29), bipolar affective disorder (F30-31) or unipolar depression (F32-33), and the patient was age 30 years or over–this is the youngest a UK Biobank participant could have been when HES began in 1996. The selection was independent of participation in UK Biobank. A dataset was produced with 100 cases with a HES diagnosis of schizophrenia, 100 with bipolar affective disorder, 100 with unipolar depression, and 50 with a wider schizophrenia spectrum disorder, with no replacement, such that each record belonged to a different person. For each of the 350 cases identified by a HES record from SLaM, all available HES records from any hospital were also extracted to a file, with their source, the primary diagnosis, and all secondary diagnoses. The researcher was able to access this file only after the validation procedure was complete, in order to extract all HES records with an F-chapter ICD-10 diagnosis, which would include admissions to SLaM hospitals, other mental health trusts, and general hospitals where a mental disorder had been included in the record.

Once the procedures below were set, it was decided that assessment of 250 cases (out of the 350) would be sufficient to draw conclusions on overall validity. These cases were chosen at random, and the clinical assessors had no access to the file of HES diagnoses, in order to ensure blinding to the HES diagnosis during the research diagnosis procedure. The main clinical assessor received only the CRIS ID for each of the cases, which enabled them to access the pseuonymised version of the entire electronic patient record generated by South London and Maudsley as described above. This record would include all of the contacts of the selected cases between the years of 2006, when electronic records began, and October 2015, when the validation procedure began.

### Validation procedure

We used a comprehensive approach to extracting diagnostic information from the record, following the “gold standard” Longitudinal, Expert, All Data (LEAD) diagnostic system defined by Spitzer [19] and guidelines for reporting of validation studies for routine data.[20] This involved extracting longitudinal psychopathology and diagnostic information from the full notes, and then using the available information to determine the most likely clinical diagnosis as an ICD-10 code for the index event (the admission leading to the HES record) and as a hierarchical lifetime diagnosis. The hierarchy that we use aligns with most diagnostic manuals in prioritising schizophrenia-like disorders over affective disorders, on the basis that the former are life-long and pervasive, and may explain symptoms of illnesses further down the hierarchy.[16, 21] In our case we specified that schizophrenia had priority, with wider schizophrenia spectrum disorders next, followed by bipolar affective disorder and unipolar depression.

A psychiatrist assessor (KD) extracted data and used a semi-structured process to explore each patient’s entire CRIS/SLaM record, with a view to gain sufficient detail about the presentation of the patient to complete an operational criteria checklist. The checklist used was the enhanced OPCRIT (or OPCRIT+),[22] which is a structured clinical and research tool consisting of a form that enquires about psychopathology and other diagnostic criteria in order to give an algorithmic guide to the likely diagnostic code in ICD and DSM coding manuals. The process involved first extracting data from structured fields then reading free-text records, in particular, the assessor paid careful attention to the period before and during the admission that generated the HES record, the first and last assessments available in the notes, standardised care planning forms (the Care Programme Approach (CPA) record), and where structured fields showed a change in the diagnosis. Finally, searches were run on the full-text record with probes to identify mention of the following: diagnostic terms (schizo*, bipolar, mania, depress*); symptom terms (manic, euphoric, hallucination, delusion*, voices, thought disorder, FTD (formal thought disorder)); and important comorbidities (alcohol, cannabis, personality). OPCRIT+ was then completed and run twice–once for the “current” (i.e. index) and again for “lifetime” diagnosis. OPCRIT+ can also guide the formation of a structured abstract of the case,[23] which was assembled for 80 cases. These abstracts avoided diagnostic terms to allow a second psychiatrist to assign diagnoses without knowledge of the opinion of the treating team, and also avoided mentioning ethnicity. An example of a structured abstract and OPCRIT+ output for a hypothetical case is provided in S1 Appendix.

Both psychiatrist assessors used the OPCRIT+ output along with other details to make research-standard diagnoses of one primary disorder and unlimited secondary disorders for index and lifetime formulations. The primary diagnosis for the index admission was allocated as the diagnosis that was the main reason for admission at that time. The lifetime primary diagnosis was allocated by hierarchy of all diagnoses considered to be met at any point by an individual. The primary diagnosis was required to be a specific code or “no psychiatric disorder”, and could not be left blank or vague. If the assessor felt there was real ambiguity, the assigned diagnosis could be flagged as uncertain. Assessor KD’s formulations are used as the ‘gold standard’ research diagnoses presented in the results section.

### Analysis and statistical methods

**Fig 2:**
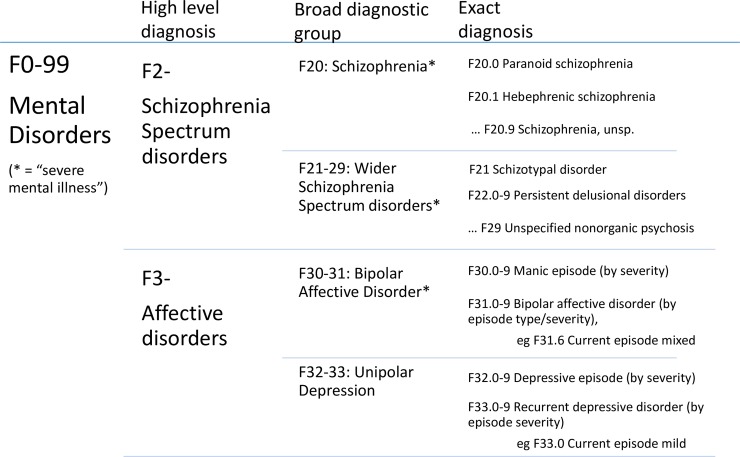
Hierarchy diagram showing the diagnoses considered in this study, with different levels of detail.

All data were entered into MS Excel, which was used for statistical analyses, graphics and random number generation. Confidence intervals around proportions were estimated using Wilson’s method [24] at 95% confidence levels. For comparing agreement between sets of diagnoses the primary measure used was the positive predictive value (PPV)–the proportion of cases identified by HES considered to be true cases of that diagnosis according to the research diagnosis. To assess the validity of single episode diagnoses for indicating the true reason for admission and/or the most serious mental disorder diagnosis for each individual we compared the index HES (the record by which the case was selected into the research) against the index and lifetime research diagnoses, and at multiple levels of detail in the diagnosis–from presence of any mental disorder, through broad diagnostic groups, to exact agreement, as shown in Fig 2.

Secondly, we test possible algorithms for extracting diagnosis or selecting cohorts from datasets where multiple HES records exist per person, and report the performance in terms of sensitivity, specificity, PPV, negative predictive value (NPV) and Cohen’s kappa, so that future researchers can select the algorithm that is optimised for their purpose (eg maximum sensitivity or highest NPV). These algorithms are largely based upon the work for Sara et al. [16]:

- "Ever": Any inpatient HES record (from mental health or not) with ICD-10 diagnosis in that diagnostic category–some individuals will have multiple diagnoses
- "More than once": Two or more HES records with a diagnosis in the range–some individuals will have multiple diagnoses
- "Last": Allocated the most recent F-code HES diagnosis, excluding F99 (a non-specific code)
- "Hierarchy": Allocated the diagnostic category received highest in the sequence above (F20 > F21-29 > F30-31 > F32-33)
- "Hierarchy > 1": Allocated the diagnostic category highest in the sequence (F20 > F21-29 > F30-31 > F32-33) of diagnoses received more than once
- "Most": Allocated the category of diagnosis that they have received most often. Where there is tie, the hierarchy rule is used

Kappa was included as a measure for overall agreement that reduces the effect of the prevalence of the disorder under study,[25] and 95% confidence intervals were calculated using the RealStatistics plug-in.[26]. For these analyses, we considered three of the individual disorder categories—schizophrenia, bipolar and depression–and the compound groups schizophrenia spectrum (schizophrenia plus wSS) and “severe mental illness” (schizophrenia spectrum plus bipolar). Schizophrenia spectrum was included instead of wSS due to the poor predictive value of wSS found when looking at the index HES.

All statistics reflect the performance of the HES diagnosis in this sample, which is people who have had an admission to a mental health provider–i.e. PPV is the proportion of people of those who had an admission to a mental health provider that resulted in a HES diagnosis of ‘condition x’ who truly had ‘condition x’; sensitivity is proportion of people with an admission to a mental health provider due to ‘condition x’ who received a HES diagnosis of ‘condition x’.

## Results

**Fig 3:**
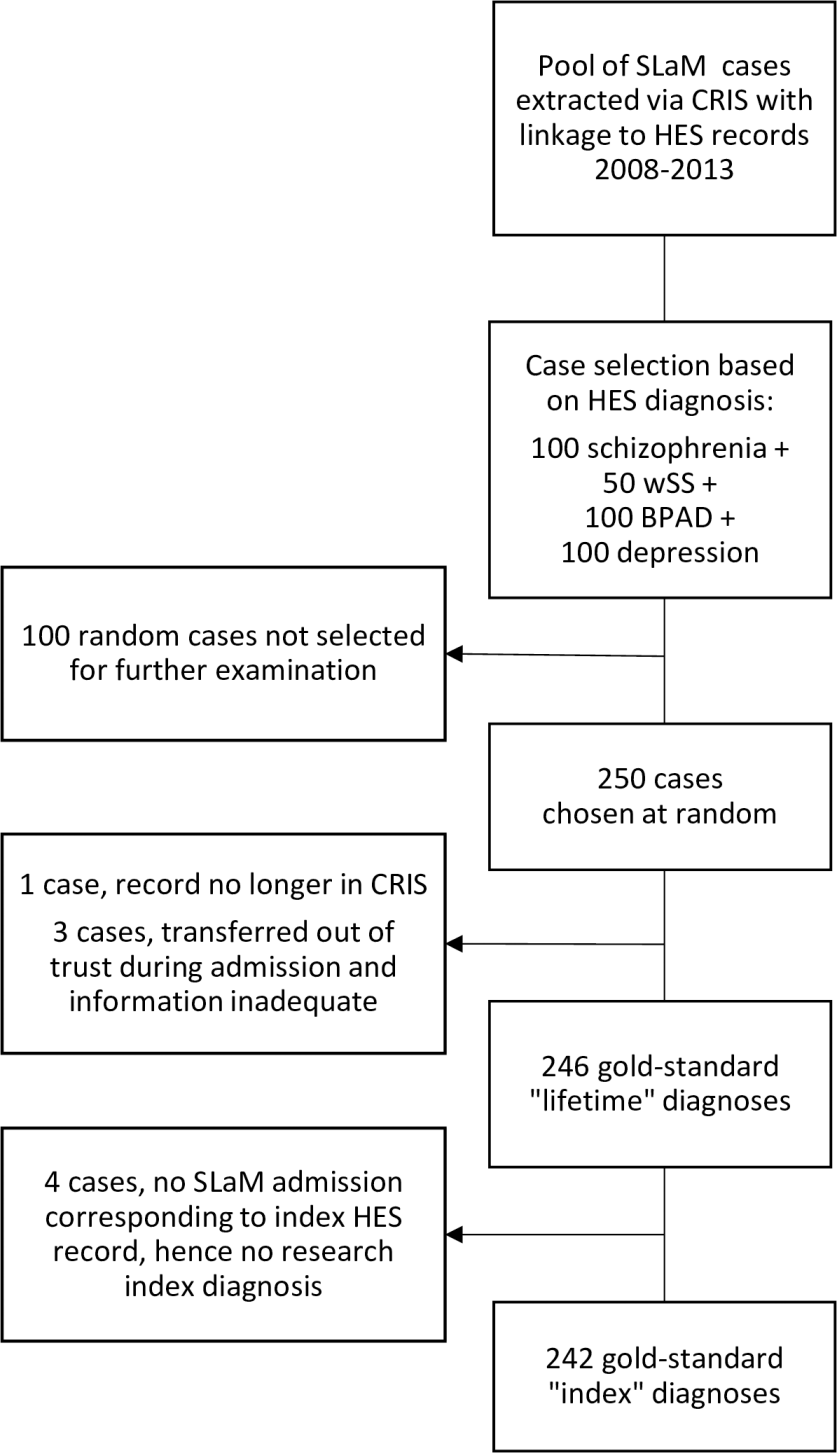
Flow chart of cases used to derive gold-standard diagnoses. Schizophrenia (ICD-10 F20); wSS = wider schizophrenia spectrum disorder (ICD-10 F21-29); BPAD = bipolar affective disorder (ICD-10 F30-31); depression = unipolar depression (ICD-10 F32-33).

Of the 250 individual cases, one was no longer in the database and three were transferred out of trust before discharge, for all of whom there was insufficient detail on which to make a diagnosis. Four cases had no SLaM admission corresponding to the HES record, but in all of these cases there were previous admissions upon which to base a lifetime research diagnosis. This meant there were 242 assessor-made “index” diagnoses and 246 “lifetime” diagnoses, as shown in Fig 3. Of the 242 index research diagnoses, 119 (49%) were marked as uncertain. In one case, there was uncertainty at the border with normality (i.e. whether the person had a mental disorder or not); in all other cases, the uncertainty concerned the boundary between disorders.

Table 1 shows the patient characteristics for the 249 cases. There were some differences between the groups, especially between the group with a HES schizophrenia diagnosis compared with a depression diagnosis on the items of race and duration of contact with services. Characteristics of those with wider schizophrenia spectrum (wSS) and bipolar diagnoses were between those of schizophrenia and depression. There is potential for some of these factors to influence the validity of the clinical (HES) diagnosis, meaning they may affect comparisons of validity between different diagnoses.

**Table 1:** Patient characteristics based on diagnosis in HES record by which they were selected.

Index HES record diagnosis:	*F20* *Schizophrenia*	*F21-29* *wSS*	*F30-31* *Bipolar affective disorder*	*F32-33* *Unipolar Depression*	Total
Number of patients	72	37	73	67	**249**
Mean age (years) (95% CI)	49 (47–51)	50 (46–55)	54 (51–56)	54 (51–57)	**52 (50–53)**
> 65 years (95% CI)	11/72 15% (8–26)	6/37 16% (7–33)	21/73 29% (19–41)	17/67 25% (16–38)	**55/249** **22% (17–28)**
male gender (95% CI)	47/72 65% (53–76)	17/37 46% (30–63)	31/73 42% (31–55)	31/67 46% (34–59)	**125/249** **50% (44–57)**
**Racial identity**					
White British (95% CI)	13/72 18% (10–29)	11/37 30% (16–47)	34/73 47% (35–59)	40/67 60% (47–71)	**97/249** **39% (33–46)**
Black (95% CI)	45/72 63% (50–73)	17/37 46% (30–63)	20/73 27% (17–39)	13/67 19% (11–31)	**95/249** **38% (32–45)**
Other (95% CI)	14/72 19% (11–31)	9/37 24% (12–42)	19/73 26% (17–38)	14/67 21% (12–33)	**57/249** **23% (18–29)**
**Service use**					
N. admissions 2006–2015: median (IQR)	3 (2–5)	3 (1–4)	3 (2–6)	1 (1–2)	**3 (1–4)**
Known for at least nine years (95% CI)	48/72 66% (53–77)	15/37 40% (24–58)	37/73 51% (39–62)	12/67 18% (10–30)	**110/249** **44% (38–51)**

Abbreviations: IQR = interquartile range, N. admissions = Number of admissions, wSS = wider schizophrenia spectrum.

Table 2 shows agreement between the index HES diagnosis and the primary research diagnoses. The strictest comparison is with index research diagnoses at three figures of the ICD-10 code (see Fig 2), where only 21% of records agreed. However, at the level of diagnostic group (i.e. schizophrenia, wSS, bipolar and unipolar depression) there was a 66% agreement in index diagnosis. Lifetime research diagnostic group agreed with index HES in 73% of cases. Agreement was highest for schizophrenia diagnosis, and lowest for wSS. Merging the schizophrenia and wSS category to make a schizophrenia spectrum category gave agreement of 86% (95%CI: 77–92). The proportion of cases for which the assessor was uncertain was highest for wSS and lowest for schizophrenia.

**Table 2:** Agreement rates for index HES diagnosis and research primary diagnosis (index and lifetime) by index HES diagnosis. Index (strict) refers to exact diagnosisprecipitating admission (3 figure ICD-10 code), Index (category) refers to diagnostic group precipitating admission, Lifetime (category) refers to diagnostic group lifetime occurrence. “Uncertain” refers to the research assessor marking the lifetime diagnosis as being uncertain.

	Index HES diagnosis				
	*F20* *Schizophrenia*	*F21-29* *wSS*	*F30-31* *Bipolar affective disorder*	*F32-33* *Unipolar Depression*	Total
n.	69	38	69	66	242
**Primary diagnosis agreement**					
-- Index (strict)	30/69 44% (33–57)	3/38 8% (2–24)	4/69 6% (2–15)	15/66 23% (14–35)	**52/242** **21% (17–28)**
-- Index (category)	59/69 86% (75–93)	13/38 33% (19–51)	46/69 67% (55–78)	42/66 64% (51–75)	**160/242** **66% (60–72)**
-- Lifetime (category)	65/72 90% (80–96)	14/38 36% (21–54)	55/72 76% (65–85)	49/67 73% (60–83)	**183/246** **73% (68–79)**
**“Uncertain”**	26/72 36%	27/38 71%	32/69 45%	37/67 55%	**122/246** **49%**

Abbreviations: wSS = wider schizophrenia spectrum disorders

The diagnoses in this study can be considered to lie on a psychosis-affective spectrum from F20 schizophrenia, through F21-29 wSS and F30-31 bipolar affective disorder, to F32-33 unipolar depression. The most common disagreement between index HES and research diagnoses was a HES diagnosis of wSS and a research diagnosis of a type of schizophrenia. The documentation of a case of schizophrenia as wSS represents a shift in the administrative record away from the psychosis end of the spectrum. Considering all discordant diagnoses (both index and lifetime) 91/145 (63%) represented a shift in the HES coded record away from psychosis diagnoses relative to the research diagnosis and 23/145 (16%) represented a shift towards psychosis. In thirty cases, the primary research diagnosis was found to be outside the diagnoses of reference, with 22/145 (16%) diagnoses of functional disorders (ICD-10 F34-F69), and 8/145 (6%) organic, neurodevelopmental or personality disorder diagnoses.

Regarding secondary diagnoses, only 15 (6%) index HES records had a secondary diagnosis. The research diagnoses had least one, often several, secondary diagnosis in 113 cases (47%), including substance use disorder in 70 (28%), a further functional diagnosis (including personality disorder) in 40 (16%), and an organic or neurodevelopmental disorder in 25 (10%). Table 3 shows that the prevalence of secondary diagnoses was fairly even across different primary diagnoses.

**Table 3:** Rate of secondary diagnoses recorded in HES and found in research diagnosis.

	Index HES diagnosis				
Primary HES diagnosis	*F20* *Schizophrenia*	*F21-29* *wSS*	*F30-31* *Bipolar affective disorder*	*F32-33* *Unipolar Depression*	Total
n.	69	38	69	66	242
***Rate of any secondary diagnoses*:**					
Index HES	5/69 7%	2/38 5%	6/69 9%	2/66 3%	**15/242** **6%**
Research diagnosis	26/69 38%	17/37 46%	36/69 52%	34/66 52%	**109/242** **45%**

Abbreviations: wSS = wider schizophrenia spectrum disorder

### Inter-rater reliability

Among the eighty cases reviewed by two assessors for index and lifetime diagnoses (160 formulations), the agreement between the two raters for primary diagnosis at the category level was 81%, with kappa 0.75 (95%CI 0.61–0.84). The degree of agreement of the assessor diagnosis with the HES primary diagnosis at the level of diagnostic group for those cases that were double reviewed was virtually identical (assessor 1: 69% kappa 0.57, assessor 2: 68% kappa 0.58). As Table 4 shows, agreement between assessors was fairly even over the different primary diagnoses, while agreement of the two assessors with HES diagnosis showed a similar pattern of agreement between diagnostic groups.

**Table 4:** Inter-rater reliability for primary diagnoses of index and lifetime formulations in 80 cases (160 formulations).

	Primary diagnosis (categories)					
	*F20* *Schizophrenia*	*F21-29* *wSS*	*F30-31* *Bipolar disorder*	*F32-33* *Unipolar Depression*	*Fx* *Other*	Total
Assessor 1 diagnoses (index and lifetime)	63	12	38	27	20	160
**Assessor 2 agreement**	51/63 81%	10/12 83%	33/38 87%	22/27 81%	14/20 70%	**130/160** **81%**
HES index diagnoses	58	10	48	44	Na	160
**Assessor 1 agreement (index and lifetime)**	49/58 84%	2/10 20%	36/48 75%	23/44 52%	Na	**110/160** **69%**
**Assessor 2 agreement (index and lifetime)**	46/58 79%	4/10 40%	33/48 69%	26/44 59%	Na	**109/160** **68%**

Abbreviations: wSS = wider schizophrenia spectrum disorder, Na = not applicable

### Multiple HES records

For the 250 individuals in the sample, all inpatient HES records were examined. There were 1015 HES records from SLaM (including the 250 index), 99 HES records (in 48 cases) from other mental health trusts and 139 HES records (in 72 cases) from general hospitals that mentioned an ICD-10 F-code diagnosis in primary or secondary diagnosis, a mean of 5.0 HES records per person. Sixty-nine (28%) people had HES records indicating a diagnosis from more than one diagnostic category. We explored algorithms that could be used with multiple records, as defined in methods.

The performance of the algorithms by diagnostic category are shown in Table 5. No single algorithm clearly performed best. Sensitivity was highest with the "Ever" algorithm, up to 93% for severe mental illness. Specificity for severe mental illness was highest in the "More than one" algorithm, at 83%, but the Hierarchy algorithms were better for specificity in depression. Agreement as assessed by kappa was maximised overall using the “last” algorithm, at kappa between 0.67 and 0.74.

**Table 5:** Accuracy statistics for algorithms to predict lifetime research diagnosis from multiple HES records, based on review of 246 cases. Numbers of cases given for each diagnostic group predicted by stated algorithm (explained in methods). PPV = positive predictive value, NPV = negative predictive value.

	*F20* *Schizophrenia*	*F30-31* *Bipolar Disorder*	*F32-33* *Unipolar Depression*	*F20-29 Schizophrenia Spectrum*	F20-31 Severe Mental Illness
Lifetime research primary diagnosis (reference)	86	51	49	117	168
Ever	96	79	75	139	188
Sensitivity	78% (69–86)	91% (81–96)	84% (72–92)	90% (84–94)	93% (88–96)
Specificity	84% (77–89)	85% (80–90)	85% (79–89)	77% (69–84)	69% (57–79)
PPV	74% (64–82)	65% (54–75)	61% (50–71)	80% (73–86)	89% (84–93)
NPV	87% (81–91)	97% (93–99)	95% (91–97)	89% (82–94)	78% (66–87)
Agree	82% (77–86)	87% (82–90)	85% (80–89)	84% (79–88)	86% (82–90)
Cohen’s kappa	0.61	0.67	0.60	0.68	0.65
More than one	73	59	42	95	147
Sensitivity	66% (56–75)	77% (64–86)	44% (32–57)	66% (57–73)	76% (69–81)
Specificity	92% (86–95)	92% (87–95)	91% (86–94)	88% (82–93)	83% (72–90)
PPV	82% (72–89)	74% (62–83)	57% (42–71)	85% (76–91)	92% (86–95)
NPV	82% (76–87)	93% (88–96)	85% (79–89)	72% (64–79)	56% (46–65)
Agree	82% (77–86)	88% (84–92)	80% (75–85)	77% (71–82)	78% (72–82)
Cohen’s kappa	0.60	0.68	0.38	0.54	0.51
Last	71	66	64	111	177
Sensitivity	71% (61–79)	84% (72–91)	83% (71–91)	82% (75–88)	91% (86–95)
Specificity	96% (91–98)	90% (85–94)	90% (85–93)	92% (86–96)	81% (70–89)
PPV	91% (82–96)	72% (60–81)	70% (58–80)	91% (84–95)	93% (88–96)
NPV	85% (79–90)	95% (91–97)	95% (91–97)	84% (77–89)	77% (66–85)
Agree	87% (82–90)	89% (84–92)	88% (84–92)	87% (82–91)	89% (84–92)
Cohen’s kappa	0.71	0.70	0.67	0.74	0.71
Hierarchy	96	49	53	139	188
Sensitivity	78% (69–86)	73% (60–82)	71% (58–82)	90% (84–94)	93% (88–96)
Specificity	84% (77–89)	96% (92–98)	93% (88–95)	77% (69–84)	69% (57–79)
PPV	74% (64–82)	85% (72–92)	73% (60–83)	80% (73–86)	89% (84–93)
NPV	87% (81–91)	92% (87–95)	92% (87–95)	89% (82–94)	78% (66–87)
Agree	82% (77–86)	91% (86–94)	88% (83–91)	84% (79–88)	86% (82–90)
Cohen’s kappa	0.61	0.73	0.64	0.68	0.65
Hierarchy >1	73	53	30	92	145
Sensitivity	66% (56–75)	73% (60–83)	40% (28–54)	64% (55–72)	75% (68–80)
Specificity	92% (86–95)	94% (90–97)	96% (92–98)	89% (83–94)	83% (72–90)
PPV	82% (72–89)	79% (66–88)	73% (55–86)	86% (77–92)	92% (86–95)
NPV	82% (76–87)	92% (87–95)	85% (80–89)	71% (63–78)	55% (45–64)
Agree	82% (77–86)	89% (85–92)	84% (78–88)	77% (71–81)	77% (71–82)
Cohen’s kappa	0.60	0.69	0.43	0.53	0.50
Most (hier)	78	67	58	116	183
Sensitivity	72% (62–80)	87% (76–94)	76% (63–85)	81% (73–87)	92% (87–95)
Specificity	92% (86–95)	91% (86–94)	92% (87–95)	88% (81–92)	75% (63–84)
PPV	83% (73–90)	74% (62–83)	72% (59–82)	87% (80–92)	91% (86–94)
NPV	85% (79–90)	96% (92–98)	93% (88–96)	82% (75–88)	77% (65–86)
Agree	84% (79–88)	90% (86–93)	88% (83–92)	84% (79–88)	87% (83–91)
Cohen’s kappa	0.65	0.74	0.66	0.69	0.67

## Discussion

We set out to determine the accuracy of certain psychiatric diagnoses as recorded in national statistics (Hospital Episode Statistics or HES) of episodes of inpatient mental healthcare. The SLaM/CRIS database of cases with electronic patient notes and associated data linkage allowed us to validate HES against a research diagnosis without needing to recontact patients. An analysis of 246 cases diagnosed with schizophrenia, a wider schizophrenia spectrum disorder, bipolar affective disorder or unipolar depression showed a perfect agreement with the presence of any mental disorder and good agreement with the presence of the stated diagnostic group of disorder (73% 68–79). When considering multiple HES records with mental disorder diagnoses, a good overall approach was to take the most recent, which showed the positive predictive value of a diagnosis was 91% for schizophrenia spectrum, 72% for bipolar affective disorder and 70% for unipolar depression. This puts the accuracy of HES records to identify the presence of a mental disorder in the range of some “objective” biomedical tests and discharge diagnoses of physical illness from general hospitals.[28] This implies that HES and similar records can be used with some confidence to indicate the likely presence of these three categories of mental disorder serious enough to require hospitalisation.

Inter-rater reliability is strong (at kappa 0.75), but considering the inter-rater reliability involved one psychiatrist researcher providing a second opinion based on the facts of the case put forward by another psychiatrist of similar background, this is lower than might be expected. There was also a high level of uncertainty in the research diagnoses (49%). This leads to a sense that some the differences between clinical and research diagnoses reflect not 'error' by the clinician or in the administrative record, but uncertainty in the true diagnosis. It is widely acknowledged that current mental disorder classifications are an imperfect representation of human psychopathology.[21, 29, 30] Out of the three main diagnoses, unipolar depression was the most uncertain, and this may be because the types of ‘depression’ that present to inpatient mental healthcare are atypical compared to the standard presentation in the community,[31] and allied with a high level of comorbidity in our sample. For bipolar affective disorder, the uncertainty seemed to be with alternative diagnoses on the schizophrenia spectrum, although there were some cases with complex personality traits and other morbidities. Schizophrenia diagnoses were accurate and stable, which was not the case with diagnoses in the wider spectrum. wSS diagnoses were often assigned to more complex cases of psychosis after some time in the service, but the validation process showed that in many cases criteria were in fact met for schizophrenia. This leads to the concept of “schizophrenia spectrum” performing well, and we would not recommend using HES records where the distinction between schizophrenia and the other schizophrenia spectrum disorders was required.

Secondary diagnoses were generally not documented in HES when present. We suspect this reflects a wide-spread tendency not to document of code for secondary diagnoses in mental health inpatients. Great caution is thus required using HES or other administrative data in evaluating mental disorders which are more likely to be coded as secondary or ‘co-morbid’–such as substance misuse, learning disability and personality disorder–as the data is likely to be unrepresentative. Specific registers may be more appropriate.[32]

Our recent review of the validity of administrative diagnoses found that there was a wide range of validity between the studies.[6] Aggregating the studies showed that the diagnoses of schizophrenia spectrum, bipolar affective disorder and unipolar depression performed similarly on PPV, with a median of 75% and a wide spread of results. Schizophrenia spectrum as a category of diagnosis performed better than schizophrenia and schizoaffective disorders separately (a large proportion of the wSS cases in this study were schizoaffective diagnoses). Our results are entirely consistent with the results of the review. With the studies ranked from lowest to highest PPV, this study is within the 25-50^th^ centile for bipolar affective disorder and unipolar depression diagnoses and within the 50-75^th^ centile for schizophrenia and schizophrenia spectrum. Our overall PPV of 73% (CI 95% 68–79) can be compared to the 13 results from studies from the review carried out using inpatient diagnoses, in which the average PPV was 77%, which is not significantly different.

### Strengths and weaknesses

Our study used a set of electronic patient records to explore patient histories in depth with patient anonymity protected through the CRIS system. We did not interview the cases under study, but it has been shown to be possible to make gold-standard psychiatric diagnoses without re-interviewing patients, where the procedure is consistent with LEAD diagnosis.[19, 33, 34] We explored index HES diagnoses from a defined service and geographic catchment in South-East London that is considered to be a centre of excellence in psychiatry, which could lead to concerns over generalisability. However, HES records came from numerous wards across four locality hospitals, providing care in a highly pressurised healthcare system for patients from various settings, including urban areas of high deprivation; therefore not untypical of mental health services elsewhere in the UK. A drawback of concentrating on a mental health trust was that we were unable to look in detail at diagnoses made in the general hospital, outpatient departments or A&E departments–which all go in to making up the entirety of HES output.

We studied three of the most prominent diagnoses for general psychiatry—schizophrenia, bipolar affective disorder and unipolar depression—covering also the whole range of what is sometimes termed “severe mental illness” by our inclusion of wider schizophrenia spectrum. However, we did not study HES diagnoses important in some psychiatric specialties, such as eating disorder and dementia, and so cannot inform questions of validity for these. We have provided a variety of outcomes (PPV, sensitivity, kappa, etc.) for a variety of algorithms to help future studies choose algorithms that are well suited to their objectives. However, the results are based on one assessor, and the inter-rater reliability showed there was some variation between assessors.

### Conclusions

Administrative health data are being used in studies such as UK Biobank to collect information on health status without the need for face to face reassessment, recontact or (in some cases) reconsent of participants. Hospital Episode Statistics (HES) in England contain ICD-10 diagnostic information, including regarding psychiatric admissions. In our study, a clinical psychiatrist assessor agreed with the HES diagnosis 73% of the time, with level of agreement varying by diagnosis. All were felt likely to have a disorder in the F chapter (mental and behavioural disorders) of ICD-10. It should be remembered that administrative data will always under-represent those who do not, or cannot, access services. Even in highly developed countries, access to services for those with mental disorder is low, at around a third.[35, 36] Our study shows that HES inpatient psychiatric diagnoses can be used, with appropriate caution, to identify cases of severe mental disorder and distinguish between some common categories of diagnosis.

## Supporting information

S1 Appendix OPCRIT+ output for hypothetical case (abstract and OPCRIT results). (DOCX)
