# Supplementary material for: Using data linkage to electronic patient records to assess the validity of selected mental health diagnoses in English Hospital Episode Statistics (HES)
Source: PLoS One. 2018 Mar 26;13(3):e0195002. doi: 10.1371/journal.pone.0195002 (PMC5868851; doi:10.1371/journal.pone.0195002)
Supplement: S1 Appendix — (DOCX) [file pone.0195002.s001.docx]

Supporting information

# Appendix S1: OPCRIT+ output for hypothetical case

## Abstract:

DEMOGRAPHICS AND PRESENTING COMPLAINT

64 year old married man presents walking down the road semi-naked claiming to be sent from God.

Currently self-employed and living in owner occupied accommodation.

Strange behaviour in last week. Previous episodes of similar behaviour, but not for many years. Recently had complained of low mood, poor energy, poor sleep, poor concentration.

FAMILY HISTORY

There is a family history of mental illness, specifically his father had

- Major depressive disorder

- Drug or alcohol dependence

died of alcohol-related disease when pt was 25.

There is no known family history of physical illnesses.

PERSONAL HISTORY

Premature birth. Normal development. Schooling disrupted when parents separated. Left without qualifications.

Worked in a bank, but was recently made redundant, and now trying to set up business from home.

Practicing Christian evangelical.

One year ago had stroke and seriously ill in hospital. Made good physical recovery, but continued to be troubled by fatigue.

PAST PSYCHIATRIC HISTORY

Six previous admissions with elated, disinhibited behaviour and religious delusions, usually under section - responding quickly to treatment. Note religious at baseline, but different quality. Triggers have been problems at work and poor sleep. Two previous suicide attempts (poisoning). No episodes in last ten years, discharged from services. Stopped maintenance medications after stroke.

PAST MEDICAL HISTORY

Past medical history of-

- Hypertension

- Diabetes (type 2, tablet controlled)

- Hyperlipidaemia

- Cerebrovascular disease

Ischaemic CVA twelve months before this episode.

DRUG AND ALCOHOL HISTORY

Ex-cigarette smoker. 15 pack-years.

history of ?harmful use of-

- Alcohol

- Cannabis

ALCOHOL HISTORY-

Age of onset of regular alcohol use was 15.

Has not drunk for many years. Previous ?misuse, but no dependence.

CANNABIS HISTORY-

Age of onset of regular Cannabis use was 15. On an average week uses approximately £0-£9 worth of cannabis.

Smokes 1-2 spliffs 'herbal' cannabis per week - finds it relaxing. Increased in the week prior to presentation ?exact amount.

OPIATE HISTORY-

No history of harmful misuse of opiates.

STIMULANT HISTORY-

No history of harmful misuse of stimulants.

MEDICATION HISTORY

Usually compliant. Had been without medication for 12 months prior to admission, as stopped when he had a stroke, as had been symptom free for 10 years.

No psychotropic medication is currently being prescribed. History of response to antipsychotics and mood stabilisers. History of 'switching' on antidepressants.

FORENSIC HISTORY

There is no known history of criminal or violent behaviour.

Contact with police only in the context of mental health.

SOCIAL HISTORY

Married with one child. Occupationally and socially capable between episodes.

PERSONALITY

There is an enduring pattern of inner experience and behaviour that deviates markedly from the expectations of the culture. This is manifested in...

...ways of perceiving and interpreting self, other people, and events

The pattern is inflexible and pervasive across a broad range of personal and social situations.

The onset of this pattern is in adolescence.

Tendency towards bizarre beliefs and experiences

Tendency towards preoccupation with perfection, order and routine

MENTAL STATE EXAMINATION

APPEARANCE AND BEHAVIOUR

The following abnormalities in appearance were noted-

- gross lack of self care

- a mildly increased BMI

The following abnormalities in behaviour were noted-

- Bizarre behaviour

- Excessive activity

- Reckless activity

- Distractibility

- Agitated activity

SPEECH AND FORM OF THOUGHT

The following abnormalities of speech and thought form were elicited-

- Positive formal thought disorder

- Pressured speech

- Thoughts racing

MOOD, AFFECT AND ASSOCIATED FEATURES

The following abnormalities of affect and mood were elicited-

- Inappropriate affect

- Irritable mood

- Dysphoria

- Poor concentration

- Increased sociability

- Increased self esteem

No complaints of suicidal ideation.

The following disturbances in sleep pattern were noted-

- Early morning waking

The following disturbances in appetite, eating behaviour and body image were noted-

- Increased appetite for 1 month or more.

- Mild weight gain recently.

ANXIETY, TRAUMA AND ASSOCIATED FEATURES

The following anxiety symptoms, were elicited, reaching time threshold for diagnostic significance-

- Autonomic arousal symptoms during times of anxiety

- Prominent, excessive, free-floating anxiety and suffers from... (always anxious. since stroke, worse)

- ...restlessness, feeling keyed-up or on-edge

- ...being easily fatigued

- ...poor concentration or 'mind going blank'

- ...irritability

- ...muscle tension

- ...sleep disturbance

- Obsessions and/or compulsions present... (cleanliness and cleaning rituals)

- ...that are repetitive and unpleasant

- ...that are resisted unsuccessfully by the subject

- ...that are acknowledged by the subject as originating in their own mind

- ...that are recognised as excessive or unreasonable

THOUGHT CONTENT

On this assessment affective symptoms predominate although psychotic symptom also sometimes occur.

Has been receiving messages through TV and other media that god wants him to go out and warn people about the end of the world, and cleanse them of their sin. Feels guilty that can't do this as well as he would like.

The following delusions, reaching time threshold for diagnostic significance, were elicited-

- Grandiose delusions

- Delusions of guilt

No passivity phenomena elicited on this assessment.

PERCEPTIONS

Hearing screaming and other noises that he thinks are damned souls.

COGNITION, INSIGHT AND CAPACITY

Cognition was not tested on this assessment.

Insight absent.

Capacity to consent to hospital admission was felt to be absent at this assessment.

Capacity to consent to medical treatment was felt to be impaired at this assessment.

Outcome

Responded to reinstatement of medication after around one week. Went home on leave, but felt overwhelmed and took an overdose. Returned back to the ward for another month. After discharge, felt able to continue work, and did this for a number of years before retiring. Discharged from services around the same time, with no contact in the last two years.

## OPCRIT Results:

ICD10 criteria: F30.2 Mania with psychosis

ICD10 criteria: F12.1 Harmful use of cannabis

DSM3 criteria: Mania with psychosis

DSM3R criteria: Bipolar disorder

DSM4 criteria: Bipolar I disorder

Tayor & Abrams criteria: Mania

Research diagnostic criteria (RDC): Schizoaffective, bipolar

Carpenter criteria: Level 5 schizophrenia

Farmer criteria: Schizophrenia, hebephrenic-like subtype

Crow criteria: Schizophrenia, type 1 (positive symptoms)

Tsuang & Winokur criteria: Schizophrenia, undifferentiated

ICD10 criteria: F60.X personality disorder

Severe trait: Obsessive-compulsive (consider obsessive-compulsive (anankastic) PD)

For more information, see the OPCRIT+ webpages <http://sgdp.iop.kcl.ac.uk/opcritplus/>
